# Lifting Spirits: Exploring the Link Between Strength Training Intensity and Depression

**DOI:** 10.1192/j.eurpsy.2025.1350

**Published:** 2025-08-26

**Authors:** S. Ben Aissa, C. Najar, K. Razki, M. Cheour, F. F. Romdhane

**Affiliations:** 1 Razi Hospital, La Manouba, Tunisia

## Abstract

**Introduction:**

In recent years, the appealing aspect of strength training has grown beyond its physical benefits, promoting interest in its potential impact on mental health. Despite the curiosity, the association between it and depression remains unexplored.

**Objectives:**

The purpose of this study is to examine at the link between strength training intensity and depression levels among active gym-goers in Tunisia.

**Methods:**

This is a cross-sectional study, conducted from February to March 2024. Participants were recruited online through social media platforms (Tunisian facebook groups and fitness forums) using a posted survey link. We’ve included respondents who are 18 years of age or older who have been active in strength training with a gym membership for 1 month or more. The respondents were required to answer a questionnaire that included questions related to socio-demographic data and to provide strength training intensity related details (sessions frequency, duration, perceived overall intensity using likert scale)

Depression levels were measured using the Patient Health Questionnaire-9 (PHQ-9).

**Results:**

The overall number of participants was 72, with 86% being male. The majority of responders (n=65, 90.2%) indicated that they performed strength training exercises at least three times per week, with an average session length of 45 minutes. In terms of strength training intensity, 38.8% (n= 28) of participants reported high-intensity sessions, 48.6%(n=35) moderate-intensity sessions, and the remaining participants reported low-intensity sessions.

The mean depression score on the PHQ-9 scale was 4.3 (SD = 2.1). Implying a prevalence of minimal to mild depressive symptoms among the research population.

Strength training intensity was found to have a negative correlation with depression scores (r = -0.36, p = 0.03), suggesting that higher intensity sessions were linked with fewer symptoms of depression.

**Conclusions:**

This study serves to shed light upon the association between strength training intensity and depression levels among Tunisian gym-goers. The findings emphasise the positive impact of strength training with higher intensity, on depressive symptoms

**Disclosure of Interest:**

None Declared

